# Numerical and functional defects of blood dendritic cells in early- and late-stage breast cancer

**DOI:** 10.1038/sj.bjc.6604018

**Published:** 2007-10-09

**Authors:** A Pinzon-Charry, C S K Ho, T Maxwell, M A McGuckin, C Schmidt, C Furnival, C M Pyke, J A López

**Affiliations:** 1 Dendritic Cell and Cancer Laboratory, Queensland Institute of Medical Research, Brisbane, Queensland, Australia; 2 School of Medicine, University of Queensland, Brisbane, Queensland, Australia; 3 Mater Medical Research Institute, Brisbane, Queensland, Australia; 4 Wesley Medical Centre, Brisbane, Queensland, Australia; 5 Department of Surgery, Mater Misericordiae Hospital, Brisbane, Queensland, Australia; 6 Australian Centre for Vaccine Development, Brisbane, Queensland, Australia

**Keywords:** dendritic cells, breast cancer, immune function, immunotherapy CD40

## Abstract

The generation of antitumour immunity depends on the nature of dendritic cell (DC)–tumour interactions. These have been studied mostly by using *in vitro*-derived DC which may not reflect the natural biology of DC *in vivo*. In breast cancer, only one report has compared blood DC at different stages and no longitudinal evaluation has been performed. Here we conducted three cross-sectional and one one-year longitudinal assessments of blood DC in patients with early (stage I/II, *n*=137) and advanced (stage IV, *n*=36) disease compared to healthy controls (*n*=66). Patients with advanced disease exhibit markedly reduced blood DC counts at diagnosis. Patients with early disease show minimally reduced counts at diagnosis but a prolonged period (1 year) of marked DC suppression after tumour resection. While differing in frequency, DC from both patients with early and advanced disease exhibit reduced expression of CD86 and HLA-DR and decreased immunostimulatory capacities. Finally, by comparing a range of clinically available maturation stimuli, we demonstrate that conditioning with soluble CD40L induces the highest level of maturation and improved T-cell priming. We conclude that although circulating DC are compromised by loco-regional and systemic breast cancer, they respond vigorously to *ex vivo* conditioning, thus enhancing their immunostimulatory capacity and potential for immunotherapy.

Dendritic cells (DC) are antigen-presenting cells (APC) that play a central role in initiating and directing cellular and humoral responses including antitumour immunity (Banchereau *et al*, 2000). These APC play a clinically important role in the defence against cancer as increased numbers of tumour-infiltrating DC correlate with better clinical prognosis (Zeid and Muller, 1993; Lespagnard *et al*, 1999). Tumours, however, employ numerous mechanisms to evade immune elimination including DC suppression (Pinzon-Charry *et al*, 2005b). Assessing the *in vivo* effects of tumours on DC is difficult and limited by their paucity in peripheral blood (Ho *et al*, 2002) and tissues. Hence, DC–tumour interactions have been addressed mostly by using DC derived *in vitro* from CD34^+^ or monocyte precursors following culture with cytokines (Menetrier-Caux *et al*, 1998; Sombroek *et al*, 2002; Peguet-Navarro *et al*, 2003). Nonetheless, cytokine-driven activity may not reflect the functional status of DC circulating *in vivo* and thus detailed evaluation of blood DC at different stages of tumour progression is necessary.

Circulating DC play a critical role in shaping antitumour responses by continually replenishing the pool of tissue-residing DC (Banchereau *et al*, 2000). These cells can be identified by their high levels of HLA-DR and lack of specific lineage markers (CD3, CD14, CD19, CD20, CD56 and CD34) expressed on other leucocytes (Thomas *et al*, 1993b; Savary *et al*, 1998). Few reports have assessed the effects of tumours on blood DC *ex vivo* (Gabrilovich *et al*, 1997; Hoffmann *et al*, 2002; Ratta *et al*, 2002; Della Bella *et al*, 2003) possibly due to their scarcity and heterogeneous nature. In the case of breast cancer, circulating DC have been shown to be numerically reduced (Della Bella *et al*, 2003), exhibit increased rates of apoptosis (Pinzon-Charry *et al*, 2005c), and reduced capacity to stimulate T cells (Gabrilovich *et al*, 1997; Satthaporn *et al*, 2004). The DC compartment has also been shown to exhibit increased number of immature cells with impaired antigen-presenting capacity (Pinzon-Charry *et al*, 2005a, 2005d). To date, however, only one – rather small – study has compared blood DC at different stages of disease (Gabrilovich *et al*, 1997) and no longitudinal studies have been reported.

The aim of this study was to monitor the blood DC compartment in patients with early- and late-stage breast cancer to ascertain any correlation between DC abnormalities and burden of disease, and identify factors suitable for clinical implementation to improve DC function. Cross-sectional and longitudinal assessments were performed in 173 patients with locally limited or metastatic disease, and results compared with 66 healthy controls. Given the low frequency of blood DC and restricted blood volumes that could be taken from patients, sub-cohorts were used for the various analyses aiming to (i) estimate blood DC numbers and their correlation with other blood counts, (ii) assess the immunostimulatory profile of blood DC and (iii) identify factors suitable for clinical implementation to functionally improve blood DC.

## MATERIALS AND METHODS

### Study subjects and design

A total of 173 female patients, 40–83 years of age, with histologically confirmed breast adenocarcinoma were enrolled in the study. Of these, 137 patients presented with early disease, either local stage I (T_1_N_0_M_0_; *n*=78) or nodal stage II (T_2_N_1_M_0_; *n*=59) and 36 patients presented with advanced metastatic stage IV (TNM_1_). In addition, 66 age-matched, female, healthy donors volunteered for the study and served as controls. All 137 patients with early-stage disease were newly diagnosed. Of the 36 patients with advanced disease, six were newly diagnosed and 30 presented with recurrence after a disease-free interval and no prior therapy for at least 7 months (range of 7–127 months, mean of 60 months). Staging was performed in accordance with the International Union Against Cancer (UICC) TNM classification (Sobin and Wittekind, 2002). Blood samples were collected prior to therapy except for the longitudinal study where in addition to the sample collected 2 weeks prior to surgery, follow-up samples were collected at 6, 24 and 48 weeks post-surgery. The 6 and 24 weeks samples were collected before and immediately after completion of the radio/chemotherapy regimens, respectively. For the longitudinal follow-up, 40 patients (all receiving hormone therapy) were divided into those receiving hormone therapy and either radiotherapy (*n*=9), chemotherapy (*n*=11) or combined therapy (*n*=20). Three stage I patients (*n*=3) not receiving hormone therapy (yet administered radiotherapy) were also included for comparison. The Australian Red Cross Blood Service provided buffy coats. Written informed consent was obtained from all patients and the study was approved by the Human Ethics Committees of clinical (Mater Health Services and Wesley Medical Centre) and research (Queensland Institute of Medical Research) institutions.

### Monoclonal antibodies, reagents and media

The following monoclonal antibodies were used: CD3, CD4, CD19, CD20, CD56, CD34, HLA-DR, CD40, CD80, CD83, CD86, CD11c, CD123 and IgG1, IgG2a and IgG2b isotype controls from BD Pharmingen (BD Biosciences, San Jose, CA, USA); HLA-DR, CD19 and IgG1 isotype control from Beckman Coulter (Fullerton, CA, USA) and IL-12 from Caltag Laboratories (Burlingame, CA, USA). All antibodies were used as fluorescein isothiocyanate (FITC), phycoerythrin (PE), biotin, allophycocyanin (APC) or PE-Cy5 conjugates. Complete media included RPMI 1640 supplemented with 10% foetal calf serum (FCS), penicillin (100 U ml^−1^), streptomycin (100 *μ*g ml^−1^), L-glutamine (2 mM), HEPES (25 mM) and nonessential amino acids, all purchased from Gibco Life Technologies (Gaithesburg, MD, USA). The combination of pro-inflammatory cytokines consisted of IL-1*β* (10 ng ml^−1^), IL-6 (10 ng ml^−1^) and TNF-*α* (10 ng ml^−1^) obtained from R&D systems (Minneapolis, MN, USA), plus prostaglandin E_2_ (PGE_2_, 1 *μ*g ml^−1^) from Sigma (St Louis, MI, USA). Double-stranded RNA (polyI:C; 50 *μ*g ml^−1^) and lipopolysaccharide (LPS; 50 ng ml^−1^) were purchased from Sigma. The CpG oligodeoxynucleotide 2216 (CpG; 3 *μ*g ml^−1^) was acquired from Geneworks (Melbourne, Victoria, Australia). The soluble human recombinant trimetric CD40 ligand (CD40L; 2 *μ*g ml^−1^) was kindly provided by Amgen (Seattle, WA, USA). Tetanus toxoid (TT) obtained from CSL (Melbourne, Victoria, Australia) was conjugated to FITC (FITC-TT) in-house and dialysed in phosphate-buffered saline (PBS) for 48 h before use. Dialysis membranes (Polylabo, Strassbourg, France) with an MW cutoff of under 10 000–14 000 were used. FITC-dextran was obtained from Sigma and sheep red blood cells from Equicell (Melbourne, Victoria, Australia).

### Flow cytometry

Following venous blood collection in heparinised tubes, peripheral blood mononuclear cells (PBMC) were isolated by Ficoll-Hypaque centrifugation. Cells were stained with the lineage (Lin) mixture (CD3, CD14, CD19, CD20, CD56 and CD34, all FITC) and HLA-DR (PE-Cy5). Three or four colour flow cytometry (FACS) was used to evaluate DC numbers, phenotype, antigen uptake and cytokine secretion. Blood DC were defined as lineage (Lin) negative and HLA-DR positive events (Lin^−^HLA-DR^+^ cells). Dendritic-cell subsets were identified using CD11c (APC) and CD123 (PE). Antigen uptake was assessed after cells were incubated (10^7^ cells ml^−1^; 60 min) with FITC-TT (0.5 mg ml^−1^) or FITC-dextran (1 mg ml^−1^) at either 4°C or 37°C. Antigen capture was calculated as the difference in mean fluorescence intensity (ΔMFI) between the test (37°C) and the control (4°C). Phenotypic maturation was assessed after culture (10^7^ cells ml^−1^) for 18–36 h in the presence of a combination of inflammatory cytokines (IL-1*β*, IL-6, TNF-*α* and PGE_2_; CC), poly I:C (PIC) or CD40L (in addition to IFN-*γ* and IL-1*β*). Where indicated, culture supernatants were collected and assayed using an IL-12 p70 ELISA kit (Mabtech AB, Nacka Strand, Sweden). Intracellular cytokine staining was performed by stimulating cells as above in the presence of brefeldin-A (10 *μ*g ml^−1^, Sigma). Cells were labelled, fixed with 1% paraformaldehyde and stained with anti-IL-12-PE in 0.2% saponin/PBS at 4°C overnight. In all experiments, 5−10 × 10^5^ events were collected within the mononuclear gate. Absolute DC counts (10^6^ l^−1^) were calculated from the number of PBMC per litre of blood (as determined by the automated cell counter) multiplied by the percentage of DC (mean of triplicates) determined by FACS analysis. Routine differential blood count measurements (10^9^ l^−1^) were performed using automated cell counters (Advia 120, Haematology System or Technicon H.3 RTX Bayer, Tarrytown, NY, USA). All data were acquired on an FACS calibur flow cytometer and analysed using CellQuest 3.1 (BD Biosciences) or Summit (Cytomation, Fort Collins, CO, USA) software.

### Cell purification and culture

Following isolation of PBMC, cells were stained with lineage mixture (FITC), HLA-DR (PE) and CD11c and CD123 (APC). As a viability indicator, 7AAD (Sigma) was included. Viable DC (Lin^−^HLA-DR^+^ CD11c^+^ CD123^+^ cells) were sorted (99% purity) using MoFlo Sorter (Cytomation) and resuspended in complete medium. Allogeneic T cells were purified from buffy coats by rosetting PBMC with neuraminidase-treated sheep red blood cells (>90% CD3^+^ T cells). Alternatively, CD4^+^ T cells were purified by positive immunoselection with anti-CD4 microbeads (>90% CD4^+^ T cells; Miltenyi Biotec, GmbH, Bergisch Gladbach, Germany). Varying numbers of DC from breast cancer patients were cultured with allogeneic T cells purified from healthy donors and incubated for 5 days in complete medium. At 16 h prior to harvesting, 1 *μ*Ci well^−1^ of ^3^H-thymidine was added to each well and thymidine incorporation was measured in a *β*-scintillation counter (MicroBeta Trilux Scintillation Counter, Wallac, Turku, Finland). For measurements of IFN-*γ* secretion, after 5 days in culture, supernatants were collected, pooled and assayed using an ELISA kit (Mabtech).

### Statistical analysis

Statistical evaluation of the data was performed using GraphPad Prism (GraphPad Software, San Diego, CA, USA). One-way analysis of variance (ANOVA) followed by Bonferroni's comparison test were used to analyse significance between different sub-cohorts of patients and controls, and between the pre-operative and subsequent time points in the longitudinal follow-up study. Results were considered to be statistically significant when *P*<0.05. For values of proliferation presented in Figure 3E, paired *T* tests were used.

## RESULTS

### Dendritic cell counts are reduced in advanced disease

**Figure 1:**
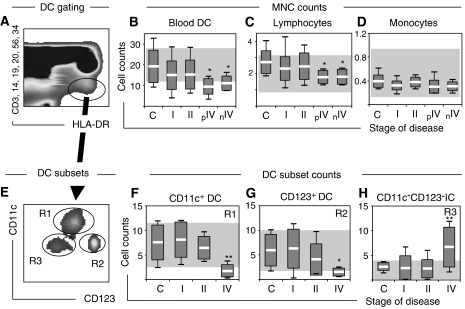
Blood DC counts at diagnosis. (**A**) A representative dot plot of PBMC from a patient with breast cancer (stage II) where blood DC are identified in the elliptical gate as lineage-negative, HLA-DR positive cells. (**B**–**D**) Mononuclear cell population counts including (**B**) DC, (**C**) lymphocytes and (**D**) monocytes were assessed in a cohort of 49 patients with breast cancer (stage I, *n*=23; stage II, *n*=11 and stage IV, *n*=15) and compared with age-matched female controls (*n*=12). Patients with advanced disease were divided into patients previously (pIV, *n*=9) or newly diagnosed (nIV, *n*=6). (**E**) Blood DC (gated as in A) in patients with breast cancer can be further analysed for DC subsets within a CD11c/CD123 bivariate plot. A representative dot plot of a patient with stage II disease is shown. (**F**–**H**) Blood DC subsets were analysed in 43 patients to determine absolute counts of (**F**) CD11c^+^DC, (**G**) CD123^+^DC and (**H**) CD11c^−^CD123^−^ immature cells. Absolute DC and DC subset counts are expressed as 10^6^ l^−1^ and all other counts are expressed as 10^9^ l^−1^. Shaded areas indicate normal reference ranges. Box plots with means, standard deviations and ranges are shown. Significant differences compared to controls are indicated as ^*^P<0.05 and ^**^P<0.01. All samples were analysed prior to therapy.

Blood DC can be identified by flow cytometry as lineage negative (Lin^−^), HLA-DR positive (HLA-DR^+^) cells (Thomas *et al*, 1993a; Figure 1A). In order to determine whether the burden of disease could have an impact on blood DC numbers in breast cancer, we estimated Lin-HLA-DR^+^ counts prior to therapy in a cohort of 49 patients grouped according to stage of disease into local (stage I; *n*=23), nodal (stage II, *n*=11) or metastatic (stage IV, *n*=15) disease, and compared these to controls (*n*=12). To limit a role for previous diagnosis/treatment in counts, analyses of early-stage disease were limited to newly diagnosed patients, and advanced disease patients were stratified into those previously diagnosed (pIV, *n*=9), thus presenting with recurrence after a disease-free interval (mean of 60 months), and those newly diagnosed (nIV, *n*=6) thus presenting for the first time. Although blood DC counts were only discretely reduced (not significant) in patients with early disease (stage I and II) compared to controls (Figure 1B), they were markedly diminished in all patients with advanced disease irrespective of their status (previously *vs* newly diagnosed), suggesting a direct effect of tumour progression on DC. The reduction in DC counts was, however, not generalised to all other mononuclear cells as lymphocytes, but not monocytes, decreased in advanced disease (Figure 1C–D). Given that the blood DC compartment has been reported to include different DC lineages including myeloid (CD11c^+^DC) and plasmacytoid (CD123^+^DC) DC (Robinson *et al*, 1999), we assessed the DC subset distribution in these patients (Figure 1E–H). There was a trend of declining CD11c^+^DC and CD123^+^DC counts with disease progression (Figure 1F–G). While CD11c^+^DC and CD123^+^DC numbers were comparable in controls and patients with local disease, they were significantly reduced in advanced disease (Figure 1F–H). We also confirmed the concurrent accumulation of immature CD11c^−^CD123^−^ cells in peripheral blood of patients with advancing disease (Figure 1H), as previously described (Pinzon-Charry *et al*, 2005a).

### Dendritic cell counts are reduced in early disease upon follow-up

**Figure 2:**
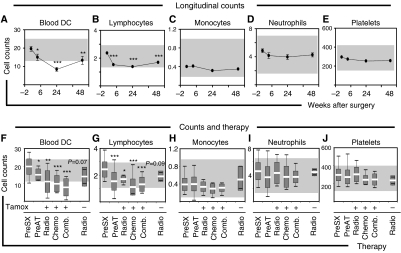
Blood DC counts follow-up. In a cohort of 40 patients with early-stage disease (stage I, *n*=26 and stage II, *n*=14), absolute counts of (**A**) DC, (**B**) lymphocytes, (**C**) monocytes, (**D**) neutrophils and (**E**) platelets were estimated at 6, 24 and 48 weeks post-surgery and compared to the pre-operative assessment (−2). (**F**–**J**) To assess the effect of the type of adjuvant therapy, the 40 patients were divided into those receiving hormone therapy (Tamoxifen, Tamox) and either radiotherapy (*n*=9; Radio), chemotherapy (*n*=11, Chemo) or combined therapy (*n*=20; Comb.). Three patients (*n*=3) not receiving hormone therapy (yet administered radiotherapy) were also included for comparison. Subsequently, (**F**) blood DC, (**G**) lymphocyte, (**H**) monocyte, (**I**) neutrophil and (**J**) platelet counts were estimated for the 24-week point (immediately after completion of radio/chemotherapy) and compared to the pre-surgery (Pre-SX, −2 weeks) and pre-adjuvant therapy (Pre-AT, 6 weeks) assessments. Note that Pre-AT corresponds to post-surgical evaluation. Absolute DC counts are expressed as 10^6^ l^−1^ and all other counts expressed as 10^9^ l^−1^. Shaded areas indicate normal reference ranges. Box plots with means, standard deviations and ranges are shown. Significant differences compared to pre-operative are shown as ^*^P<0.05, ^**^P<0.01 and ^***^P<0.001.

Given that DC counts in patients with early disease were only modestly reduced at first assessment (Figure 1), we set out to carefully monitor DC at various times over a 1-year interval in a comparable cohort of patients. Here, 40 patients with early disease (stage I, *n*=26 and stage II, *n*=14) undergoing surgery were assessed 2 weeks pre-operatively, and at 6, 24 and 48 weeks after surgery. As shown in Figure 2A, we found a progressive decline in DC counts starting as early as 6 weeks post surgery (prior to initiation of adjuvant therapy) reaching a nadir at 24 weeks (immediately after completion of adjuvant chemo- and radiotherapy) and persisting until the 48-weeks assessment. Notably, DC counts did not return to pre-operative levels in any patient (data not shown). As shown in Figure 2B, lymphocyte counts showed comparable changes to DC, decreasing significantly at 6 weeks and remaining suppressed until the 48-weeks assessment. Monocyte, neutrophil and platelet counts, however, revealed minimal changes over the matched interval (Figure 2C–E). In our previous study of healthy individuals undergoing surgery, DC counts returned to pre-operative levels within 1 week (Ho *et al*, 2001). Hence, we hypothesised that adjuvant therapy could be playing a role in the impaired DC count recovery. Because the 40 patients in our follow-up study received hormone therapy (Tamoxifen), the effect of other treatment modalities was evaluated by grouping subjects according to whether they also received chemo-, radio- or combined therapy (chemo and radiotherapy). Notwithstanding their limited number, DC counts of three patients not receiving hormone therapy (yet receiving radiotherapy) were included for comparison (labelled as radio without Tamoxifen in Figure 2F–J). In all patients, DC counts were estimated after completion of their radio/chemotherapeutic regimen (24 weeks after surgery) and compared to the pre-surgery (Pre-Sx, -2 weeks) and pre-adjuvant therapy (Pre-AT, 6 weeks) assessments. Patients on hormone therapy were on Tamoxifen at the time of evaluation. Despite the different course of therapy (local, systemic or combined), DC numbers fell significantly in all groups compared to the pre-surgery assessment (Figure 2F). Lymphocyte counts were also markedly reduced in all groups. Patients not on hormone therapy (but receiving radiotherapy) demonstrated moderate (not significant) reduction in DC and lymphocyte counts. Patients receiving local radiotherapy (with or without hormone therapy) exhibited moderately (not significant) higher counts than patients receiving chemo- or combined therapy (Figure 2G). Monocyte, neutrophil and platelet counts showed little or no alteration in either group during corresponding assessments (Figure 2H–J).

### Dendritic cells exhibit impaired immunostimulatory phenotype

**Figure 3:**
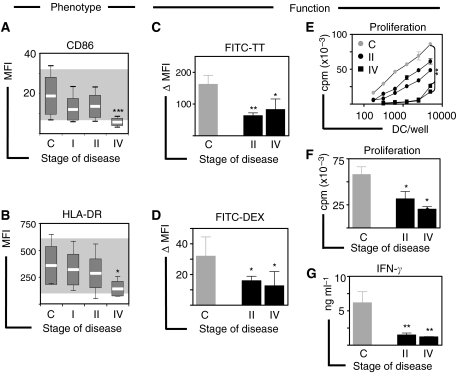
Blood DC exhibit reduced immunostimulatory phenotype. (**A**) In a cohort of 40 patients with breast cancer (stage I, *n*=19; stage II, *n*=12 and stage IV, *n*=9), the level of expression of (**A**) CD86 and (**B**) HLA-DR was analysed according to the stage of disease and compared with controls (*n*=*14*). Shaded areas indicate normal reference ranges and box plots show means, standard deviations and ranges. (**C** and **D**) Antigen uptake of soluble (FITC-TT) and particulate (FITC-Dextran) antigens by blood DC from patients with early (stage II, *n*=*7*) and advanced (stage IV, *n*=*4*) disease compared to controls (*n*=*7*) is presented as the difference in mean fluorescence intensity. (**E**) Allo-stimulatory capacity of blood DC as tested by mixed leucocyte reaction (MLR). One representative experiment in which increasing numbers of blood DC purified from patients with early (stage II, *n*=*2*) or advanced (*n*=*2*) disease and a healthy control (*n*=*1*) were tested against allogeneic T cells purified from a panel (*n*=*3*) of healthy volunteers. The pairs giving maximal responses are shown as means of triplicate measurements. (**F**) Summary of proliferation data from MLRs using DC from patients with early (stage II, *n*=6) and advanced (stage IV, *n*=*3*) disease compared to controls (*n*=13) at a 1 : 30 DC:T ratio. Similar results were found for all DC:T ratios. (**G**) Summary of IFN-*γ* secretion in culture supernatants collected from MLRs at a 1 : 30 DC:T ratio as assessed by ELISA. Error bars correspond to s.e.m. Significant differences compared to controls are shown as ^*^P<0.05, ^**^P<0.01 and ^***^P<0.001. All samples were analysed prior to therapy.

Next, we set out to determine if numerical alterations of blood DC were associated with phenotypic or functional abnormalities in early and/or advanced disease. Because expression of MHC II and costimulatory molecules on the surface of DC from cancer patients has been correlated to their immunostimulatory capacity (Gabrilovich *et al*, 1997), we evaluated the expression of HLA-DR and CD86 in 40 patients with early and advanced disease (stage I, *n*=19; stage II, *n*=12 and stage IV, *n*=9) compared to controls (*n*=14). These patients exhibited DC counts comparable to those observed in the previous cohort (mean±s.e.m.; controls: 18.7±2.7; stage I: 13.3±1.1; stage II: 13.6±1.93 and stage IV: 8.9±1.8), confirming the significant reduction in DC counts in advanced disease. In this group, DC from patients with early disease showed a discrete (not significant) reduction in the expression of CD86 and HLA-DR compared to controls, while DC from patients with advanced disease showed significantly lower levels of these molecules on their surface (Figure 3A–B). The proportion of DC expressing CD86 was also significantly (*P*<0.05) reduced in advanced disease (data not shown). To further assess DC in these patients, we tested their capacity to capture antigens and stimulate proliferation and cytokine secretion in allogeneic T cells. We found that DC from controls (*n*=4) had a significantly higher capacity to take up soluble (FITC-TT) and particulate (FITC-Dextran) antigens compared to DC from both, early (stage II, *n*=4) and advanced (stage IV, *n*=4) disease (Figures 3C–D). Moreover, DC from patients with early (stage II, *n*=6) and advanced (stage IV, *n*=3) disease induced significantly reduced proliferation of allogeneic T cells (Figure 3E–F) and were poor stimulators of IFN-*γ* secretion (Figure 3G) compared to DC from control donors (*n*=*13*). Also, to assess the impact of tumour removal on the functional phenotype of blood DC, we assessed DC counts and activation status in six patients with stage II disease before (at diagnosis) and 24 weeks after completion of therapy (24 weeks after completion of radio/chemotherapy and 48 weeks after surgery). While DC and lymphocyte numbers were markedly decreased, all other blood counts (monocytes, neutrophils and platelets) showed minimal or no alteration, in accordance with our longitudinal study. No significant changes in DC phenotype were observed after completion of therapy as assessed by CD40 and CD86 expression (Supplementary Figure 1).

### Dendritic cells respond vigorously to *in vitro* conditioning with CD40L

**Figure 4:**
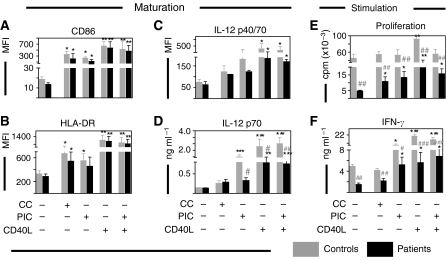
Effect of *in vitro* conditioning on blood DC from early disease. The expression of (**A**) CD86, (**B**) HLA-DR and (**C**) IL-12 was determined by flow cytometry following incubation (24 h) in the absence or presence of a cytokine cocktail (CC), synthetic double-stranded RNA (poly I:C, PIC) or CD40L, in blood DC from patients with early-stage breast cancer (stage II, *n*=8, black bars) and controls (*n*=6, grey bars). In parallel, (**D**) IL-12 p70 secretion was determined by ELISA in culture supernatants. Unstimulated or stimulated cells were cocultured for 5 days with 10^5^ allogeneic T cells from a panel of healthy donors (*n*=3). (**E**) Proliferation was determined by thymidine incorporation and (**F**) IFN-*γ* secretion by ELISA. The pairs giving maximal responses are plotted. Control (grey bars) and patients (black bars) values are presented as mean and s.e.m. Statistically significant differences between stimulated (CC, PIC, CD40L or PIC plus CD40L) and fresh samples are indicated as ^*^P<0.05, ^**^P<0.01 and ^***^P<0.001. Significant differences between breast cancer and control samples are indicated as ^#^*P*<0.05, ^##^*P*<0.01 and ^###^*P*<0.001. All samples were analysed prior to therapy.

**Figure 5:**
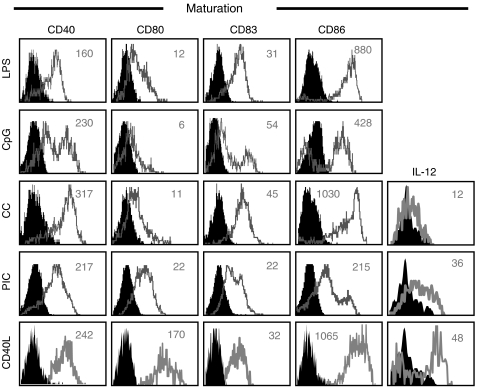
Effect of *in vitro* conditioning on blood DC from advanced disease. Response of blood DC to pro-inflammatory factors. Phenotypic maturation in blood DC from patients with advanced breast cancer (stage IV, *n*=*5*) was evaluated by assessing the expression of costimulatory molecules CD40, CD80, CD83 and CD86 and secretion of IL-12 following incubation (24 h) with a cytokine cocktail (CC), CD40 ligand (CD40L) or pathogen-derived factors, including ligands for TLR4 (lipopolysaccharide, LPS), TLR3 (polyI:C, PIC) and TLR9 (CpG). Histograms indicate expression in the absence (filled) or presence (empty) of stimulation. Numbers indicate delta mean fluorescence intensity (ΔMFI, stimulated minus unstimulated cells). Data are from two patients with advanced disease and are representative of five patients who were assessed. All samples were analysed prior to therapy.

Aiming to identify factors that could improve the function of DC from breast cancer patients in a clinical setting, we assessed the functional maturation induced by three different types of stimuli currently under investigation for immunotherapy. First, we tested (i) a combination of inflammatory cytokines (IL-1*β*, IL-6, TNF-*α* and PGE_2_; CC; Jonuleit *et al*, 1997), (ii) synthetic double-stranded RNA (polyI:C; PIC; Cella *et al*, 1999) and (iii) CD40 ligand (CD40L; Terheyden *et al*, 2000) in DC from patients with early-stage disease (stage II, *n*=8). While all types of maturation stimuli induced significant increases in the expression of CD86 and HLA-DR, CD40L was the most potent stimulus of phenotypic maturation for blood DC in these patients (Figure 4A–B). In contrast to CC and PIC, CD40L induced robust secretion of IL-12 in DC from patients, although not reaching control levels (Figure 4C–D). While DC conditioned with CC or PIC exhibited enhanced stimulatory abilities (T-cell proliferation and IFN-*γ* secretion) compared to unconditioned DC, CD40L remained the most potent stimulus to improve the immunostimulatory capacity of DC from patients (Figure 4E–F). Interestingly, addition of PIC to CD40L did not further improve the enhancement of DC induced by CD40L alone in either patients or controls (Figure 4A–F). To assess the functional maturation of DC from advanced disease patients (stage IV, *n*=5), we also tested their response to stimulation with CC and various ligands for toll-like receptors (TLR) including TLR4 (lipopolysaccharide, LPS), TLR3 (PIC) and TLR9 (bacterial oligodeoxynucleotide CpG, CpG). We found that all maturation stimuli induced significant increases in the expression of costimulatory molecules (CD40, CD80, CD83 and CD86) while only CD40 ligation evoked robust secretion of IL-12 (Figure 5).

## DISCUSSION

In the present study we have characterised the systemic effects of breast cancer on circulating DC in patients with early- and late-stage disease and identified factors that improve their function for immunotherapy. We found that blood DC numbers are only modestly reduced in early disease but are significantly reduced in patients with more advanced disease. Importantly, DC counts are consistently reduced in all patients with advanced disease irrespective of their status (previously or newly diagnosed). The marked decline in DC counts correlating with disease progression indicates an incremental effect of the tumour on blood DC populations. Previous evidence from *in vitro* studies has indicated that tumour-derived factors can affect DC differentiation from their progenitors, thus potentially reducing their numbers in peripheral blood (Gabrilovich *et al*, 1996). In fact, reduced blood DC counts have been reported in multiple myeloma (Ratta *et al*, 2002), head and neck squamous cell carcinoma (Almand *et al*, 2000; Hoffmann *et al*, 2002) and metastatic disease in colorectal, gastric, lung and renal cell carcinoma (Lissoni *et al*, 1999). In breast cancer, blood DC counts have been found decreased (Della Bella *et al*, 2003) although extensive comparative analyses of early *vs* advanced disease have not yet been described. Our results extend published data (Gabrilovich *et al*, 1997; Della Bella *et al*, 2003; Satthaporn *et al*, 2004) and demonstrate that diminished numbers of DC correlate with more extended disease.

Although patients with early disease showed only discrete (not significant) reductions in counts at diagnosis, suppression of DC was clearly evident upon follow-up. By monitoring DC counts over 48 weeks in patients with early disease, we found significant DC suppression as early as 6 weeks post-surgery extending for over 1 year with no recovery. This is notable, because apart from lymphocytes, all other blood counts (i.e. monocytes, neutrophils and platelets) showed little alteration throughout the follow-up. This is particularly relevant, given that monocytes are *in vitro* precursors of DC (Sallusto and Lanzavecchia, 1994) thought to differentiate into DC under inflammatory conditions *in vivo* (Randolph *et al*, 1999). Our data, however, indicate that blood DC and monocyte numbers are regulated differently and suggest that in cancer patients, monocyte numbers may be maintained to supply peripheral tissues, while blood DC could be specifically suppressed, as described elsewhere (Pinzon-Charry *et al*, 2005c). From an immunotherapy perspective, the finding that monocyte numbers are not affected in patients with early- and/or late-stage cancer is also relevant, because these cells are the most commonly used precursor to generate DC for therapeutic applications.

The decline in blood DC counts in patients with early disease maintained long after surgery contrasts with our previous study of healthy individuals undergoing a surgical procedure. In those patients, blood DC counts returned to pre-operative levels by day 7 (Ho *et al*, 2001), suggesting that in the current study, factors different to surgical stress were playing a predominant role. Adjuvant therapy (hormone, chemo- or radiotherapy) could be a contributing factor. Despite the different therapeutic modality, we found that patients receiving an aggressive course of systemic chemotherapy (alone or in combination with radiotherapy) had a similar decline in DC counts to patients receiving a more conservative radiotherapy regimen. Interestingly, the drop in DC numbers in all three groups (chemo-, radio- or combined therapy) coincided with a reduction in lymphocytes, but not other cell populations. This finding suggests a specific restraint on key elements of the immune response, rather than the generalised suppression of haematopoiesis expected as a consequence of antineoplastic therapy. Although a definitive conclusion is difficult to reach due to the limited number of patients available for analysis, the suggestion that adjuvant (chemo/radio) therapy appears not to exert a significant effect on numerical reconstitution of DC in patients with early breast cancer is in accordance with a previous report in a variety of cancers (Markowicz *et al*, 2002).

Despite our endeavour, a controlled assessment of the effect of hormone therapy on the abnormal DC count recovery in patients with early disease was impossible due to the absence of control cases (i.e*.,* patients with early-stage breast cancer not receiving hormone therapy). Only three patients not receiving hormone therapy (yet administered radiotherapy) were assessed. These patients demonstrated only moderate (not significant) reduction in DC and lymphocyte counts compared to pre-operative assessment possibly due to the small data set. It is plausible, however, that ongoing adjuvant anti-oestrogen therapy may have had an effect on the prolonged decrease in peripheral DC in these patients. In fact, it has been shown that anti-estrogens (such as Tamoxifen) inhibit the *in vitro* functional differentiation of human monocytes (Komi and Lassila, 2000), synovial macrophages (Komi *et al*, 2001) and murine splenic monocytes (Nalbandian *et al*, 2005) into DC, and affect basic immunological functions such as cytokine and chemokine expression (Bengtsson *et al*, 2004). However, there is no evidence as yet to suggest blood DC levels are affected by oestrogen inhibitors *in vivo*. Our results demonstrate that neither DC nor lymphocyte counts are significantly different between patients on hormone plus radiotherapy or those on radiotherapy alone. In fact, counts appear remarkably similar. A recent investigation into the function of plasmacytoid DC in female subjects showed these cells to produce more IFN-*α* than their male counterparts, a function not modified by oestrogen receptor antagonism (Berghofer *et al*, 2006). These findings are in agreement with our *in vitro* evaluation of blood DC in the presence of various equivalent therapeutic doses of Tamoxifen, showing no significant effect on DC phenotype or viability (Pinzon-Charry, *et al* unpublished observations), indicating that, at least, these features are independent of oestrogen modulation. In future, a definitive conclusion will come from the assessment of DC counts in healthy female subjects at risk of developing breast cancer and for whom Tamoxifen is recommended, thus avoiding the compounding effects of therapy and malignancy.

In our patients, it is also plausible that occult malignancy could have contributed to the abnormal DC count recovery in the longitudinal study (i.e. via tumour-derived factors or bone marrow suppression). The high rate of spontaneous apoptosis in blood DC from patients with early-stage breast cancer (Pinzon-Charry *et al*, 2005c), and the fact that early dissemination of tumour cells in bone marrow has been found in 20–40% of patients assigned to early-stage disease (Janni *et al*, 2000) support this assumption. Interestingly, we found DC were suppressed in a coordinated fashion with lymphocyte but not other blood counts. At face value, this might be interpreted as specific suppression of two key interacting components of a cognate immune response, a well-described mechanism of tumour-induced suppression (Pinzon-Charry *et al*, 2005b). In breast cancer, it has been demonstrated that lymphopenia can be completely independent of treatment modalities known to affect immune function (radio/chemotherapy) and instead appears to be a clear indicator of the host's response to tumour growth (Papatestas and Kark, 1974). While our results confirm these data, the 48-week follow-up period does not allow us to determine an association between reduced DC counts, as an index of immune competence, and tumour relapse. Prolonged follow-up of these patients (i.e. until tumour recurrence) would be necessary.

The assumption that tumour progression is associated with increasing immune cell suppression is further supported herein by the marked decrease in DC counts observed in patients with higher tumour burdens (metastatic disease). While it may be suggested that decreased DC numbers could reflect increased migration into the tumour site, previous reports indicate the contrary (Bell *et al*, 1999; Coventry *et al*, 2002). A more plausible explanation would relate to tumour-induced suppression of DC populations while in circulation. In fact, significant apoptosis of blood DC in patients with breast cancer has been reported to be associated with tumour products (Pinzon-Charry *et al*, 2005c). This phenomenon would certainly impose chronic stress on the immune system of these patients and result in progressive paucity of DC in the circulation (Lissoni *et al*, 1999; Della Bella *et al*, 2003), failure to replenish tumour-infiltrating DC and impaired migration of DC to lymphoid organs (Satthaporn *et al*, 2004) for the initiation of T-cell responses.

From a clinical perspective, the reduced functionality in blood DC from patients with early disease support the notion that DC impairment is present even in the absence of overt numerical or phenotypic alterations. The important implication for these patients is that although DC may ‘seem’ normal, their reduced functionality (Gabrilovich *et al*, 1997; Della Bella *et al*, 2003; Satthaporn *et al*, 2004) could indeed favour tumour progression by failing to induce adequate antitumour immunity. From an immunotherapy standpoint, such *in vivo* deficiencies should be overcome (Pinzon-Charry *et al*, 2006a). Here, by testing a range of clinically available maturation factors, we identified an appropriate stimulus to condition blood DC from breast cancer patients with early- and late-stage disease. Our results show that although responses in patients do not reach control values, CD40 stimulation induces a vigorous maturation and secretion of IL-12, thus generating better T-cell responses than those induced by all other factors tested. As a key molecule in the interface between T cells and DC, CD40L has long been recognised as an important element to license APC for efficient antitumour T-cell priming (Terheyden *et al*, 2000; Onaitis *et al*, 2003). However, limited reports on the clinical use of CD40L-conditioned DC for cancer immunotherapy are available (Davis *et al*, 2006). While our findings advance this reagent as one of the most potent stimuli for improving blood DC and further support its use to condition DC for breast cancer immunotherapy (Pinzon-Charry *et al*, 2006a, 2006b), its application in a clinical setting remains to be established.

In summary, this study demonstrates that the blood DC compartment is compromised in patients with early as well as late breast cancer, suggesting a correlation between DC impairment and tumour burden. While our data identify CD40L as one of the most potent stimulus to condition blood DC from patients with breast cancer, studies on the combined use of TLR ligands with pro-inflammatory cytokines demonstrate that combinatorial stimulation provide strong IL-12 secretion *in vitro* and thus may provide a better biological framework on which to develop effective DC-based immunotherapies for breast cancer.
