# Supplementary figures and images for: Numerical and functional defects of blood dendritic cells in early- and late-stage breast cancer
Source: Br J Cancer. 2007 Oct 9;97(9):1251–9. doi: 10.1038/sj.bjc.6604018 (PMC2360464; doi:10.1038/sj.bjc.6604018)

## Slide 1
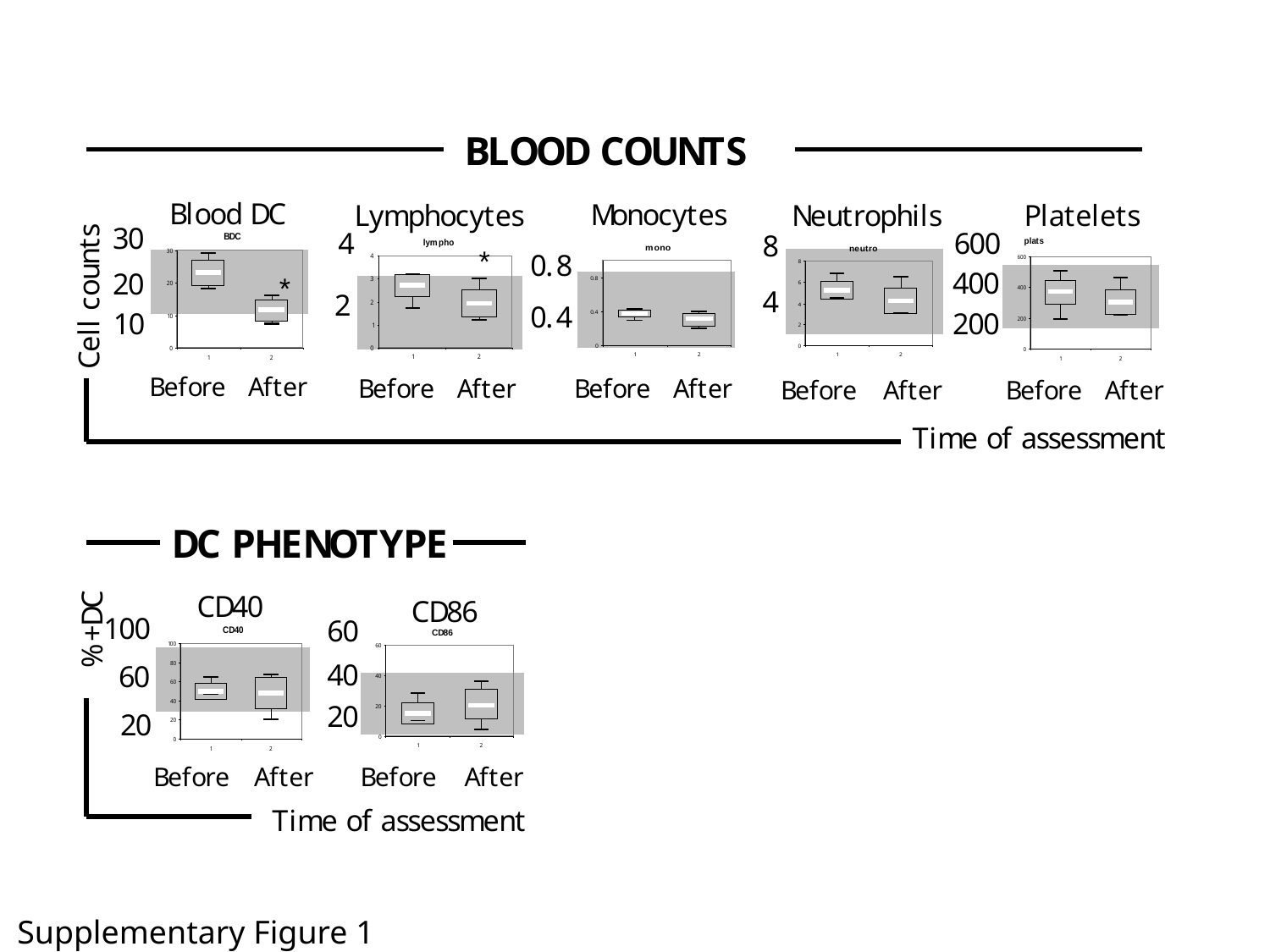

Supplementary Figure 1

Supplement: Supplementary Figure 1 [file 6604018x1.ppt]
