# Supplementary material for: Numerical and functional defects of blood dendritic cells in early- and late-stage breast cancer
Source: Br J Cancer. 2007 Oct 9;97(9):1251–9. doi: 10.1038/sj.bjc.6604018 (PMC2360464; doi:10.1038/sj.bjc.6604018)
Supplement: Supplementary Figure 1 Legend [file 6604018x2.doc]

**Supplementary Figure 1**

**Effect of tumour removal on blood counts and phenotype**

In a group of 6 patients with early stage disease (Stage II, *n*=6), absolute counts of DC, monocytes, lymphocytes, neutrophils and platelets were estimated at diagnosis (before) and 24 weeks after completion of therapy (24 weeks after completion of radio/chemotherapy and 48 weeks after surgery). Absolute DC counts are expressed as 10^6^/L and all other counts expressed as 10^9^/L. The mean percentage of Lin^-^HLA-DR^+^ blood DC expressing CD40 and CD86 are also shown (% ^+^DC). Shaded areas indicate normal reference ranges and box plots show means, standard deviations and ranges. Significant differences between ‘before’ and ‘after’ assessments are shown as **p*<0.05.
